# Effects of sample storage and shell orientation on LA-ICPMS trace element measurements on deep-sea mussels

**DOI:** 10.1038/srep17793

**Published:** 2015-12-08

**Authors:** Luciana Génio, Klaus Simon, Steffen Kiel, Marina R. Cunha

**Affiliations:** 1 Departamento de Biologia & CESAM – Centro de Estudos do Ambiente e do Mar, Universidade de Aveiro, 3810-193 Aveiro, Portugal; 2 Geoscience Center, Department of Geochemistry, University of Göttingen, Goldschmidtstr. 5, 37077 Göttingen, Germany; 3 Geoscience Center, Geobiology Group, University of Göttingen, Goldschmidtstr. 3, 37077 Göttingen, Germany; 4 Department of Palaeobiology, Naturhistoriska riksmuseet, Box 50007, 10405 Stockholm, Sweden

## Abstract

Geochemical markers are being increasingly applied to fundamental questions in population and community ecology in marine habitats because they allow inferences on individuals dispersal, but vital effects, small sample size and instrumental limitation are still challenging particularly in deep-sea studies. Here we use shells of the deep-sea bivalve *Idas modiolaeformis* to assess potential effects of sample storage, mineralogy, and valve orientation on LA-ICPMS measurements. Trace element concentrations of ^24^Mg, ^43^Ca, ^88^Sr, ^137^Ba, ^208^Pb, and ^238^U are not affected by the two most commonly used storage methods of biologic deep-sea samples (frozen at –20°C and fixed in 95% ethanol); thus combined analysis of differently preserved specimens is possible when the number of individuals is insufficient and distinct sample fixation is needed for multiple purposes. Valve orientation had a strong impact on quantification of trace elements in the calcitic but not in the aragonitic layer of adult shells. Hence, to enable comparisons between adult shells and entirely aragonitic embryonic shells, a reference map of site-specific signatures can potentially be generated using the aragonitic layer of the adult shells. Understanding ontogenetic changes and environmental effects in trace element incorporation is critical before geochemical fingerprinting can be used as a tool for larval dispersal studies in the deep-sea.

The trace element and stable isotope compositions of biogenic carbonates formed by marine organisms are widely used to understand ocean biogeochemistry, climate history, ocean acidification, and anthropogenic pollution, among a wide range of other topics^1^,^2^,^3^,^4^,^5^,^6^,^7^. A promising recent development are ‘natural tag’ or ‘fingerprinting’ studies to investigate dispersal pathways of marine larvae, contributing to the understanding of population connectivity patterns that are fundamental to biodiversity conservation and marine spatial management^8^,^9^. Tracing marine larval dispersal is challenging due to the small size of the larvae and the vastness of the ocean. The fingerprinting approach takes advantage of calcified structures, such as fish otoliths or bivalve shells that preserve the local geochemical signatures throughout the life cycle of the organism^8^. Bivalves form a calcified embryonic shell at or very close to their natal origin, then undergo a larval dispersal phase, and finally secrete a calcified shell as benthic adults, while retaining their embryonic and larval shells in the apical regions of the juvenile and adult shells. The trace element composition of the embryonic shell thus provides a ‘natural tag’ that allows inferences about the natal origin and hence larval dispersal distance of individual specimens^8^.

Challenges of geochemical marker applications include different biological effects on trace element incorporation between embryonic and adult shells^10^,^11^ as well as biases induced by preservation and storage methods^12^,^13^,^14^,^15^. In this study, we explore the applicability of the elemental fingerprinting approach using chemosymbiotic deep-sea mussels (Bivalvia, Mytilidae, Bathymodiolinae). Similar to their shallow-water relatives, which are abundant and widely distributed ecosystem engineers within intertidal rocky shores, bathymodiolins are foundation species in deep-sea chemosynthesis-based communities worldwide^16^,^17^.

Like most mytilid mussels, bathymodiolins have two early ontogenetic phases; first a non-feeding embryo that secretes a D-shaped shell of 40–80 microns width (often called prodissoconch I, or PD1), which then develops into a plankton-feeding pelagic veliger larva that secretes an up to 500 micron wide prodissoconch II shell (PD2)^18^. After settlement, the mussel becomes sessile and forms the adult shell (dissoconch). While the prodissoconchs I and II are composed entirely of aragonite, the adult shell is bimineralic, consisting of a calcitic outer fibrous prismatic layer and a nacreous aragonitic inner layer^19^,^20^,^21^. Because there are differences in the incorporation of trace elements into the crystal lattices of aragonite and calcite^22^,^23^, and therefore ontogenetic differences between larval and adult mussel shells, Becker *et al.*^24^ pointed to the limited use of adult shells to generate a reference map of site-specific signatures and assign natal origin of mussel larvae. Yet, distinguishing trace element signatures between mytilid shell layers has been overlooked, and previous studies focused on comparisons either between early larval shells or between post-settled juveniles and adults^25^,^26^,^27^.

Here we analyse the geochemical signatures of embryonic and adult shells of *Idas modiolaeformis* collected from a sunken wood log at the Gorringe Bank (NE Atlantic) with an overall focus on methodological issues. Our specific aims are (i) to assess the effect of sample orientation on the measured element concentrations of adult shell layers, (ii) to investigate potential biases induced by sample storage (frozen vs. ethanol), and (iii) to assess the potentiality of this species for larval tracking studies.

## Materials and Methods

### Specimen collection and preparation

The deep-sea mussel species *Idas modiolaeformis* was the dominant coloniser of a sunken wood log collected with the ROV *Hercules* (dive H1202, 36°38.57 N, 11°36.19 W, 1296 m depth, 14/10/2011) during E/V *Nautilus* cruise NAO17 at the NW side of Gettysburg Seamount, Gorringe Bank (NE Atlantic). Onboard, the wooden log was divided into four segments, two of which were kept inside aseptic plastic bags at −20 °C, and the other two were transferred to plastic containers filled with 95% ethanol. The samples (i.e. wooden logs and attached macrofauna) were kept in these conditions for a period of eight months. Prior to analyses, mussels were removed from the wood surface of both ethanol-preserved and frozen samples, photographed and measured under a binocular microscope. Valves were split open with a ceramic-coated razor blade and soft tissues were removed and kept for later molecular identification. Valves were transferred to Teflon beakers and immersed in 15% H_2_O_2_ (Rotipuran; P-LAB a.s.) buffered with 0.05 mol.L^−1^ NaOH (Tritipur; Merck Millipore) for approximately 10h to remove organic matter from the shell, including the periostracum. Valves were then rinsed three times in Milli-Q water and mounted on a petrographic slide against double sided tape using a wet paintbrush and Milli-Q water. Mounted valves were dried overnight in a C-100 laminar flow hood.

### Elemental analysis of mussel shells

**Figure 1:**
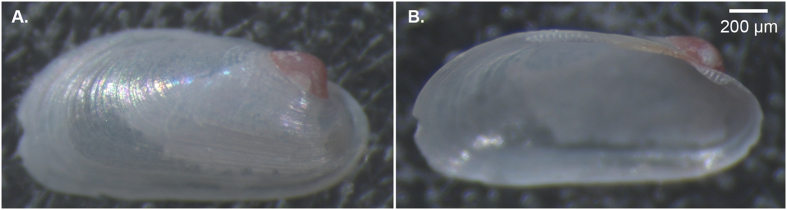
*Idas modiolaeformis* shell valves mounted with inversed orientation. (**A**) Rv- outer surface facing upwards, and (**B**) Lv-inner surface facing upwards.

In order to assess the concentration and distribution of trace elements in the mussel shell and the potential effects of sample orientation, measurements were conducted on inversely mounted valves of an adult mussel specimen (Fig. 1): the right valve was oriented with the outer surface facing upwards (so that the calcitic layer was analysed first), and the left valve inverted, with the inner surface upwards (so that the aragonitic layer was analysed first). On each valve, measurements were taken at nine spots along the entire length of the dissoconch shell (1.8 mm), and the shells were perforated (and analysed) through their entire thickness. For the assessment of the storage method effect, we compared the geochemical signatures of 10 ethanol-preserved (mean shell length 3.36 ± 0.71 mm) and 10 frozen specimens (mean shell length 3.41 ± 0.64 mm). Valves were all mounted with the inner surface upwards, which also exposes the outer surface of the prodissoconch (PD1). Measurements were made on the PD1 (one spot) and dissoconch (three spots).

Continuous ablation of shell material was performed with a 3 Hz pulsed ArF-Excimer Laser (Compex 110, Lambda Physik, Göttingen, Germany) with 193 nm wavelength (5 J cm-2 energy, spot size 40 μm). The ablated material was transported via an Ar-flow into the inductively coupled plasma (ICP) ion source of a quadrupole mass spectrometer (Perkin Elmer Elan DRC II, Canada). Eleven isotopes (^24^Mg, ^27^Al, ^29^Si, ^43^Ca, ^55^Mn, ^57^Fe, ^66^Zn, ^88^Sr, ^137^Ba, ^208^Pb, ^238^U) were analysed for 10 ms each resulting in a complete mass spectrum of 1-second time; 200 spectra were recorded. The measured intensities were transformed into concentrations by applying an external calibration with NBS610 (NIST, USA) reference material, and an internal standardization using ^43^Ca as the internal standard isotope. The adhesive tape and the slide were analysed without mussel samples to assess the risk of contamination by the tape or slide material that might be ablated and included in the analysis.

### Statistical analysis and data processing

**Figure 2:**
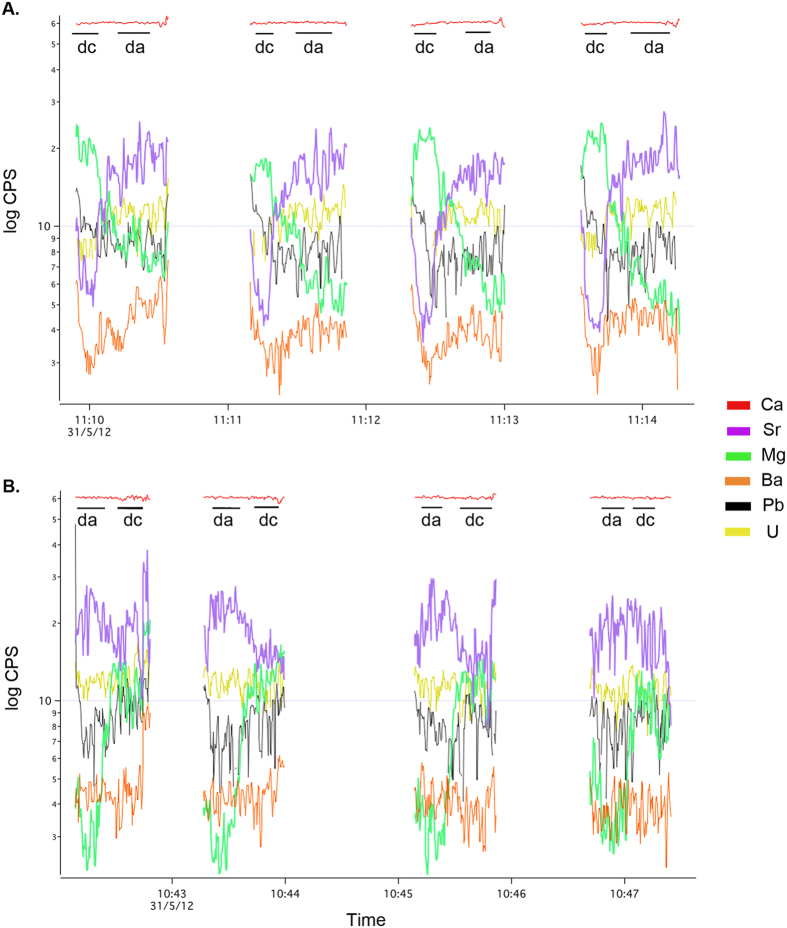
Mass spectra of six elements from *Idas modiolaeformis* shell valves with inversed orientation, (**A**) Rv-calcite facing upwards, and (**B**) Lv-aragonite facing upwards, showing integration intervals corresponding to calcitic (dc) and the aragonitic (da) shell layers.

Mass spectra were visualized with Iolite v2.15^28^, a non-commercial freeware solution implemented as a self-contained package running in the Igor Pro v6.34A (Wavemetrics Incorporated, Portland, Oregon, USA) environment. Concentration values were obtained by integrating the results in defined time intervals. On each dissoconch spot profile, two integration intervals were defined based on the switching intensities of Mg and Sr, corresponding to the transition between the calcitic and the aragonitic shell layers; namely, higher Mg and lower Sr quantities for calcite, and *vice-versa* for aragonite (Fig. 2). One integration interval (20–70 seconds) was obtained from each PD1 isotope spectrum. Box and whiskers plots for each element ratio (X: ^43^Ca) were generated using the software GraphPad Prism v6.0 (GraphPad Software Inc., San Diego, California, USA). Comparisons of multi-element signatures between mineral layers (calcite and aragonite) of the two valves, and between storage methods (frozen vs ethanol) of the prodissoconch and dissoconch shells, were analysed with principal coordinates ordination (PCO) followed by permutational multivariate analysis of variance (PERMANOVA), with “mineral layer”, “valve orientation”, “storage method” and ontogenetic “shell stage” as fixed factors, each with two levels. When significant differences were found (P(perm) < 0.05, number of permutations = 9999), *a posteriori* pairwise comparisons were also examined (within “valve orientation”, between “mineral layers”; between “valve orientation”, within “mineral layers”; between “valve orientation”, between “mineral layers”). Multivariate (all elements) and univariate (single elements) analyses were conducted on normalized data and resemblance matrices were calculated based on Euclidean distance, using PRIMER v6.1.13^29^ with the add-on PERMANOVA+^30^.

## Results and Discussion

### Effect of shell orientation

**Figure 3:**
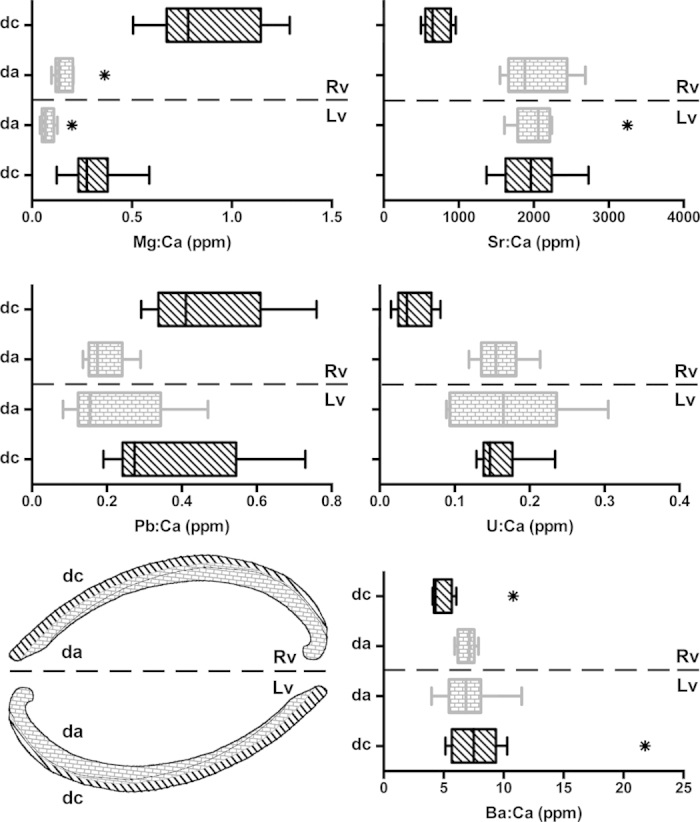
Element concentrations (X:Ca, parts per million) in calcitic (dc, black diagonal strikes) and aragonitic (da, grey brick pattern) layers of the dissoconch shell, on inversely mounted valves: Rv-calcite facing upwards, Lv-aragonite facing upwards. Bottom left: sketch showing the different shell layers in different shell orientations. Vertical lines: median value; boxes: interquartile range (IQR); whiskers: values <1.5 × IQR. Asterisks denote outliers (>1.5 × IQR).

**Figure 4:**
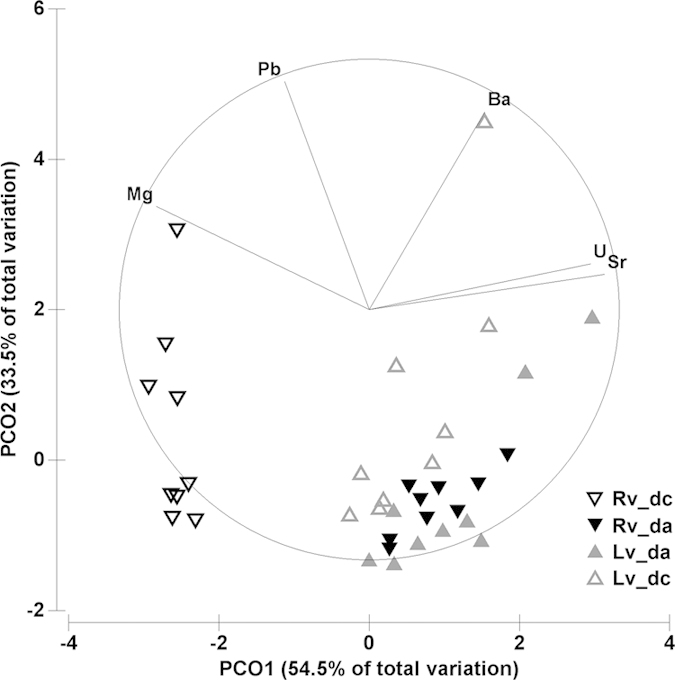
Principal coordinate (PCO) analysis of multi-element signatures in calcitic (dc) and aragonitic (da) layers of the dissoconch shell, on inversely mounted valves: RV-calcite facing upwards, LV-aragonite facing upwards (see sketch in Fig. 2).

Out of the eleven analysed isotopes, six (^24^Mg, ^43^Ca, ^88^Sr, ^137^Ba, ^208^Pb, and ^238^U) provided reliable measurements in the dissoconch shell of the deep-sea mussel *Idas modiolaeformis*. Two elements, ^27^Al and ^57^Fe gave clearly erroneous readings (e.g. negative values) and were eliminated from the analysis^31^. Because the amounts of ^66^Zn, ^55^Mn and ^29^Si in the tape and the slide were twice as high as in the shell samples, these isotopes were also removed from further analyses. Figure 3 compares the concentration values of the elements in the calcitic and aragonitic layers of two valves in relation to “valve orientation”. When the shell was oriented with the outer shell upwards (calcite ablated first, Fig. 2A) the trace element compositions of the calcitic and aragonitic layers were significantly different (Table 1). The calcitic layer is significantly enriched in Mg and Pb and depleted in Sr, U and Ba when compared to the aragonitic layer (Fig. 4). However, in the valve with the inner shell upwards (aragonite ablated first, Fig. 2B), element signatures of the calcitic and aragonitic layers could not be distinguished, except for Mg concentration. In this case, measurements in the calcitic layer differed with “valve orientation”, often significantly, while measurements in the aragonitic layer were similar irrespective of “valve orientation” (Fig. 3, Table 1).

Distinguishing calcitic and aragonitic layers in mytilid adult shells is crucial when trace element data are being compared between adult and embryonic or larval shells because the latter are entirely aragonitic. Our study indicates that bias can potentially be induced by the orientation of the shell during measurements. In agreement with previous studies^22^,^23^, magnesium concentrations were always higher in the calcitic layer, independent of shell orientation, thus allowing easy distinction of the two mineralogies in the mass spectra obtained by LA-ICPMS. However, significant differences in the concentrations of all other trace elements were detected when the calcitic (outer) layer was ablated and measured first, but not when the nacreous (inner) layer was measured first. This suggests that the ablation of aragonitic nacreous layer caused contamination on measurements of the calcitic layer, while there was little or no contamination from the ablation of calcite on measurements of the aragonitic layer. Further work is needed to clarify if this is due to the different mineralogies of the two shell layers, but this may be explained by the particular ultrastructure of nacre. Nacre is composed of stacks of minute (ca. 1 × 10 × 10 micron) plates enveloped in an organic matrix^32^. These tiny plates, or fragments of them, may be more easily eroded during ablation, thus contaminating subsequent measurements of the calcitic layer. On the other hand, the more densely packed fibrous prisms composing the calcitic layer are unlikely to contaminate subsequent ablation of the nacre. As a result, the magnesium spectrum is a reliable indicator of mineralogy when selecting the intervals for measurement integration, but quantification of other elements of distinct mineral layers must take into account the orientation of the sample.

However, measurements of trace element concentrations in the aragonitic layer (nacre) of the dissoconch shells were nearly identical regardless of which layer was measured first. This is useful for comparisons between adult (aragonitic layer) and embryonic shells because laser ablation measurements of the prodissoconch I of *Idas modiolaeformis* (and likely other mytilid species due to similar shell morphologies) is facilitated by positioning the shell with the inner (aragonitic) side facing upwards; this orientation exposes the umbonal region allowing vertical laser shots on the outer surface of the embryonic shell. Measurements can thus be taken from both prodissoconch and dissoconch shells without the need to re-orient the specimen.

### Storage method

**Figure 5:**
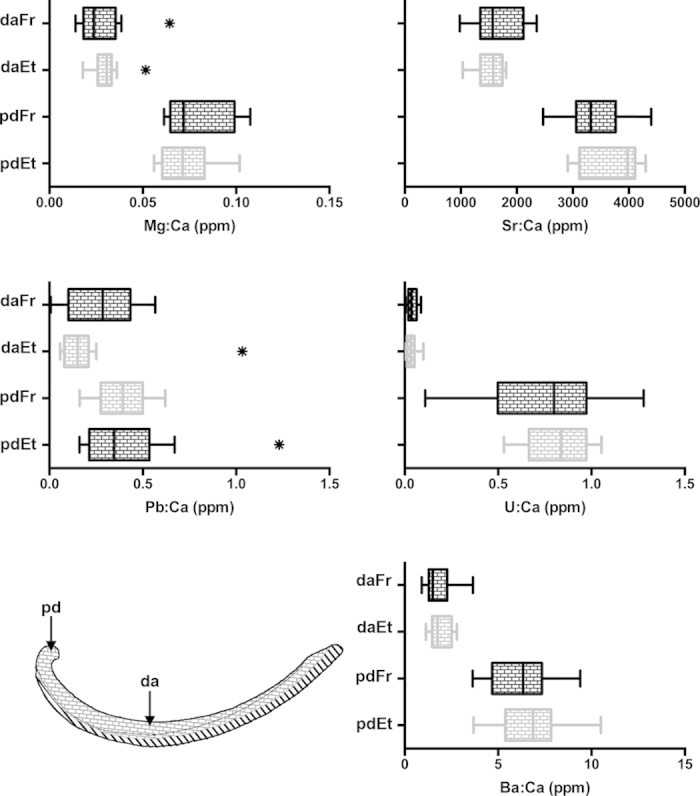
Element concentrations (X:Ca, parts per million) in prodissoconch (pd) and dissoconch (da) shell regions (sketch of valve cross section in bottom left corner of figure) in specimens preserved in ethanol (Et, grey brick pattern) and frozen (Fr, black brick pattern). Vertical lines: median value; boxes: interquartile range (IQR); whiskers: values <1.5 × IQR. Asterisks denote outliers (>1.5 × IQR).

**Figure 6:**
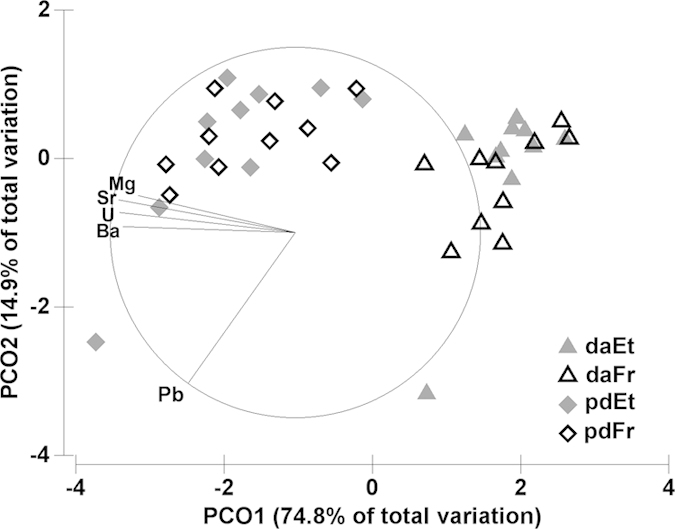
Principal coordinate (PCO) analysis of multi-element signatures in prodissoconch (pd) and dissoconch (da) shell regions (see sketch in Fig. 4) in specimens preserved in ethanol (Et) and frozen (Fr).

The storage method (frozen at −20 °C vs 95% ethanol) had no significant effect on the quantification of Mg, Sr, Pb, U and Ba in prodissoconch and dissoconch shells of *Idas modiolaeformis* (Table 2), either considering individual elements analysed separately (Fig. 5) or multi-element signatures (Fig. 6). Measurements obtained from differently preserved specimens can thus be analysed together with a negligible risk of biasing the results. This is promising and particularly relevant in deep-sea organisms because sampling is often limited to a small number of individuals, and splitting of samples into various fixatives is commonly required for multiple methodological applications (e.g. molecular and morphological studies). Previous reports on the effects of sample handling and storage on fish otoliths and deep-sea coral microchemistry identified small but measurable effects on the concentrations of some trace elements including Ba, in frozen and ethanol preserved samples, respectively^12^,^15^. However, in both cases the differences were small or negligible relative to instrument precision, or to the variability between the otolith’s core and edge. Swan *et al.*^14^ also observed small differences between pairs of treated otoliths but the variation attributable to storage and handing effects was smaller than between-geographic area differences.

### Applicability to larval tracking studies

The same five elements (Mg, Sr, Pb, U and Ba) were measured in the embryonic (aragonitic) shells (prodissoconch I) of *I. modiolaeformis* and showed significantly higher concentrations than in the aragonitic layer of dissoconch shells (Figs 5 and 6). This may either reflect different physicochemical conditions in the environment where the embryonic and adult shells precipitated, or indicate that ontogenetic changes in vital effects influence the incorporation of trace elements into the shell. While the simple enrichment in all five trace elements intuitively suggests changing vital effects - possibly in adults there is an increased discrimination ability against ions that are not calcium or carbonate - these relationships are not straightforward for all elements^11^. This has implications on the use of dissoconch shells to generate site-specific elemental signatures because putative vital effects interfere with abiotic conditions limiting the assignment of other recruited individuals to their natal origin. An alternative is to cultivate larvae *in situ* to generate site-specific larval signatures that can be compared with larval shells of naturally settled juveniles^24^. However, transplanting larvae in deep-sea habitats is extremely challenging owing to time and financial constraints and technical difficulties of *in vitro* larval rearing^18^. Moreover, empirical evidence points towards ontogenetic vertical migrations of planktotrophic bathymodiolin larvae, allowing long-distance dispersal in surface waters for periods as long as one year^33^, but it is unknown how far the developing embryos drift from their natal source during the ~8 days-long embryonic stage, when they secrete the prodissoconch I shell. This poses additional challenges to determine the natal origin of deep-sea mussel larvae. Further research is needed to elucidate the influence of abiotic factors and biologically mediated processes controlling trace element incorporation in larval and adult shells of deep-sea mussels.

## Additional Information

**How to cite this article**: Génio, L. *et al.* Effects of sample storage and shell orientation on LA-ICPMS trace element measurements on deep-sea mussels. *Sci. Rep.* **5**, 17793; doi: 10.1038/srep17793 (2015).

## Figures and Tables

**Table 1:** Multivariate and univariate results of PERMANOVA *a posteriori* pairwise comparisons of element concentrations between shell “mineral layers” (da - dissoconch aragonite, dc – dissoconch calcite) in relation to “valve orientation” (Rv - calcite facing upwards (outer surface), Lv – aragonite facing upwards (inner surface), refer to sketch on Fig. 3).

	All Elements	Mg:Ca	Sr:Ca	Pb:Ca	U:Ca	Ba:Ca
*Within “valve orientation”, between “mineral layers”*						
Rv_da, Rv_dc	6.404****	7.537****	8.433****	4.779****	8.792****	2.118*
Lv_da, Lv_dc	1.363^ns^	4.676***	0.650^ns^	1.948^ns^	0.359^ns^	0.993^ns^
*Between “valve orientation”, within “mineral layers”*						
Rv_da, Lv_da	0.520^ns^	2.599*	0.304^ns^	0.473^ns^	0.407^ns^	0.053^ns^
Rv_dc, Lv_dc	3.737****	5.651****	8.332****	1.167^ns^	8.184****	1.884*
*Between “valve orientation”, between “mineral layers”*						
Rv_dc, Lv_da	5.078****	8.662****	8.152****	3.410**	4.423****	1.594^ns^
Rv_da, Lv_dc	1.608*	2.671*	0.357^ns^	2.769**	0.0800^ns^	1.095^ns^

^****^P(perm) < 0.0001; ***P(perm) < 0.001; **P(perm) < 0.01; *P(perm) < 0.05; ns: not significant.

**Table 2:** Effect of storage method (−20 °C versus 95% ethanol) on the geochemical compositions of ontogenetic shell stages (prodissoconch and dissoconch) based on PERMANOVA.

		All Elements		Mg:Ca		Sr:Ca		Pb:Ca		U:Ca		Ba:Ca	
Source	df	MS	Pseudo-F	MS	Pseudo-F	MS	Pseudo-F	MS	Pseudo-F	MS	Pseudo-F	MS	Pseudo-F
Shell stage	1	125.2	66.05****	28.73	104.3****	32.49	196.0****	4.086	4.253*	30.98	141.8****	28.93	105.1****
Stor. method	1	0.333	0.175ns	0.070	0.255ns	0.068	0.413ns	0.002	0.002ns	0.073	0.332ns	0.12	0.434ns
Stage x Stor.	1	1.2	0.633ns	0.28	1.016ns	0.475	2.868ns	0.327	0.340ns	0.084	0.383ns	0.035	0.127ns
Residuals	36	1.896		0.275		0.166		0.961		0.218		0.275	
Prodissoconch													
Stor. method	1	3.606	0.710ns	0.907	0.902ns	1.872	1.967ns	0.150	0.143ns	0.374	0.362ns	0.304	0.292ns
Residuals	18	5.077		1.005		0.952		1.047		1.035		1.038	
Dissoconch													
Stor. method	1	1.536	0.296ns	0.180	0.172ns	0.906	0.901ns	0.209	0.200ns	0.038	0.036ns	0.202	0.194ns
Residuals	18	5.192		1.046		1.005		1.044		1.053		1.044	

df = degrees of freedom; MS = mean sum of squares; Pseudo-F = F value by permutation;

****P(perm) < 0.0001; *P(perm) < 0.05; ns: not significant.
